# Supplementary material for: Altered Mucosal Immune-Microbiota Interactions in Familial Adenomatous Polyposis
Source: Clin Transl Gastroenterol. 2022 Mar 16;13(7):e00428. doi: 10.14309/ctg.0000000000000428 (PMC10476795; doi:10.14309/ctg.0000000000000428)
Supplement: Supplementary file 3 [file ct9-13-e00428-s003.pdf]

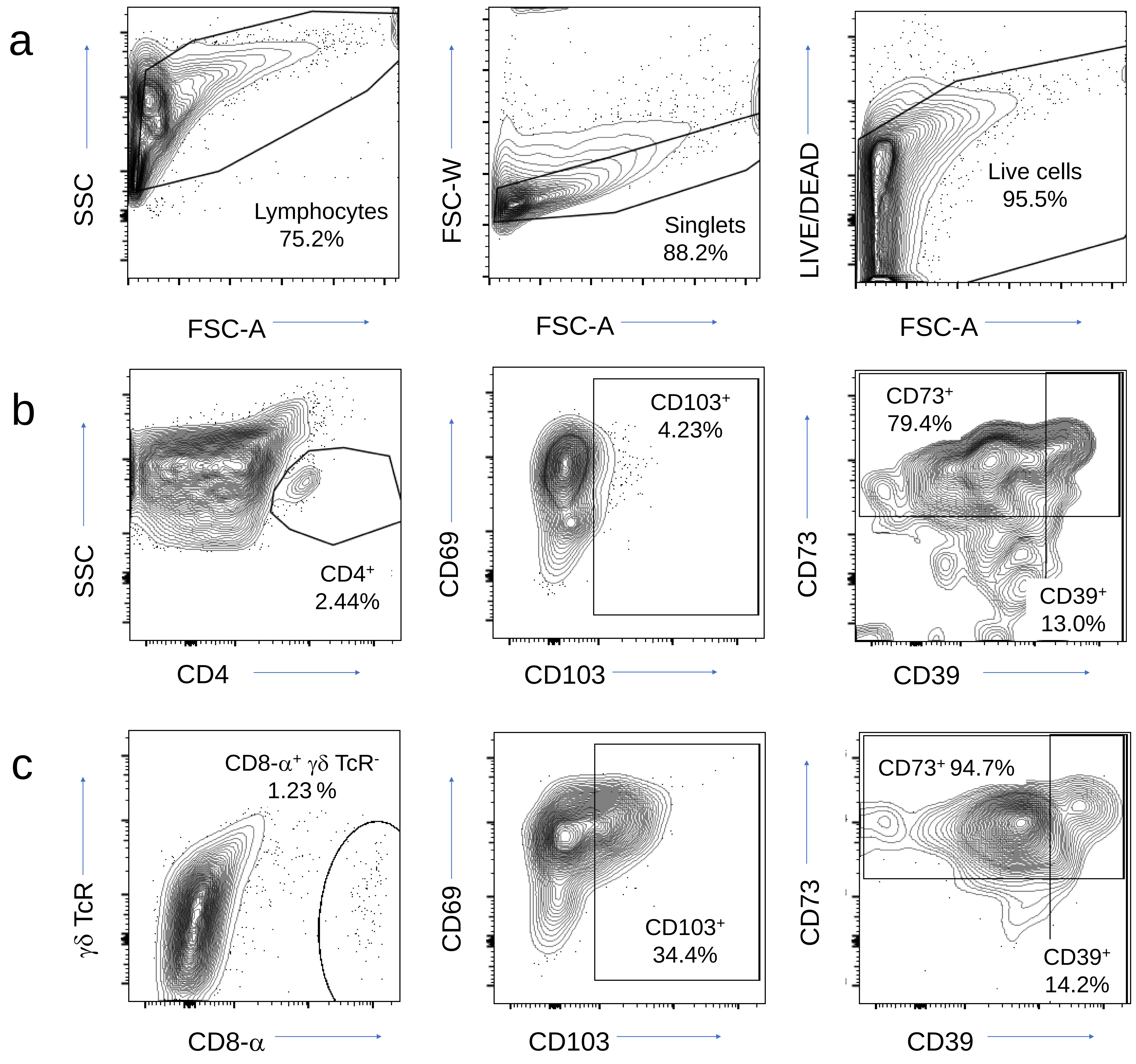

Supplementary Figure 2. LPL gating strategies. **a**. Sequential gates for live lymphocytes. **b**: Sequential gates for $CD4^+$ $CD103^+$ Trm expressing CD39 & CD73. **c**: Sequential gates for $CD8^+$ $CD103^+$ Trm expressing CD39 & CD73.
